# Online Public Attention Toward Premature Ejaculation in Mainland China: Infodemiology Study Using the Baidu Index

**DOI:** 10.2196/30271

**Published:** 2021-08-26

**Authors:** Shanzun Wei, Ming Ma, Xi Wen, Changjing Wu, Guonian Zhu, Xiangfu Zhou

**Affiliations:** 1 Department of Urology The Third Affiliated Hospital Sun Yat-Sen University Guangzhou China; 2 Department of Urology West China Hospital Sichuan University Chengdu China; 3 Andrology Laboratory West China Hospital Sichuan University Chengdu China; 4 Institutes for Systems Genetics, Frontiers Science Center for Disease-Related Molecular Network West China Hospital Sichuan University Chengdu China

**Keywords:** premature ejaculation, Baidu Index, infodemiology, public interest, patients’ concern, sexuality, sexual dysfunction

## Abstract

**Background:**

Premature ejaculation (PE) is one of the most described psychosocial stress and sexual complaints worldwide. Previous investigations have focused predominantly on the prospective identification of cases that meet researchers’ specific criteria. The genuine demand from patients with regard to information on PE and related issues may thus be neglected.

**Objective:**

This study aims to examine the online search trend and user demand related to PE on a national and regional scale using the dominant major search engine in mainland China.

**Methods:**

The Baidu Index was queried using the PE-related terms for the period of January 2011 to December 2020. The search volume for each term was recorded to analyze the search trend and demographic distributions. For user interest, the demand and trend data were collected and analyzed.

**Results:**

Of the 36 available PE search keywords, 4 PE searching topics were identified. The Baidu Search Index for each PE topic varied from 46.30% (86,840,487/187,558,154) to 6.40% (12,009,307/187,558,154). The annual percent change (APC) for the complaint topic was 48.80% (*P*<.001) for 2011 to 2014 and –16.82% (*P*<.001) for 2014 to 2020. The APC for the inquiry topic was 16.21% (*P*=.41) for 2011 to 2014 and –11.00% (*P*<.001) for 2014 to 2020. For the prognosis topic, the annual APC was 11.18% (*P*<.001) for 2011 to 2017 and –19.86% (*P*<.001) for 2017 to 2020. For the treatment topic, the annual APC was 14.04% (*P*<.001) for 2011 to 2016 and –38.83% (*P*<.001) for 2016 to 2020. The age distribution of those searching for topics related to PE showed that the population aged 20 to 40 years comprised nearly 70% of the total search inquiries (second was 17.95% in the age group younger than 19 years). People from East China made over 50% of the total search queries.

**Conclusions:**

The fluctuating online popularity of PE searches reflects the real-time population demands. It may help medical professionals better understand population interest, population concerns, regional variations, and gender differences on a nationwide scale and make disease-specific health care policies. The internet search data could be more reliable when the insufficient and lagging registry data are completed.

## Introduction

Premature ejaculation (PE) is one of the most described psychosocial stress and sexual complaint worldwide [1]. The estimated PE prevalence rate is 16% and over 20% in an internet-based survey [2,3]. Characterized with poor controlled and rapid ejaculation, the PE condition negatively impacts both sexual partners with symptomatic psychological problems and humanistic economic burdens [2,4]. However, instead of seeking help from professionals, previous reports revealed that most patients with PE are prone to initiate information-seeking behavior by using the internet due to embarrassment and wrong beliefs [5,6]. The PE prevalence and its actual impact may hence remain unveiled and underestimated.

The quality and liability of online health care–related content have been previously examined and scrutinized. Though the content was mainly acceptable, the amount of misleading content and their impact on the audience cannot be ignored. Currently, *searching before seeing a doctor* has become a trend in patients. The inquiry frequency and concerning problems from the users have been well documented by internet platforms and their demographic data. Using data from these internet platforms, infodemiology research has been successfully practiced in reporting disease incidence [7,8], surveilling pandemic outbreaks [9,10], and analyzing other public health events and related public awareness [11,12]. Previously, public interest and the change over time of the search volume in sexual dysfunction were analyzed [13,14]. It was shown that consulting *Dr Internet* influences the decision-making process of inquisitive people. Additionally, the extent of misinformation penetration could not be neglected [6,13]. However, such data in mainland China is still lacking.

By 2020, the netizens population has reached 940 million in mainland China [15]. With the 766 million users actively seeking medical information and inquiring about symptoms for diagnosis confirmation on the internet, we believe it is crucial to evaluate users search behavior and their interest or beliefs [16]. As the leading search engine in mainland China, Baidu has taken 92.1% of the search volume and 93.1% coverage of the use rate exclusively [17]. Baidu’s big data analyzing platform, Baidu Index, enables users to track the popularity change and correlated demand of one specific phrase [7,8]. Therefore, we aim to evaluate the online search trend and user demand related to PE problems and the genuine needs from a *real-world* geospatial and temporal database.

## Methods

### Keyword Selection and Data Retrieval

This study was mainly referring to the temporal search trends of PE-related terms in Chinese. The PE keyword was identified by referring to the International Society for Sexual Medicine’s definition [1]. To reduce the language habit–derived differences and biases, all possible synonyms or complex derivatives were screened and selected as previously described [8,18]. According to the available search keywords, four categories of PE topics were identified. All included PE search keywords were checked on the Baidu Index platform for their availability and are listed in Multimedia Appendix 1.

In the trend module, the Baidu Search Index (BSI) value is provided for each keyword at the national and subnational scale [8]. The BSI value is a numerical value that represents the sequential search volume on a daily basis. Hence, each keyword’s monthly search index values from January 1, 2011, to December 31, 2020, were collected at the national and provincial level from the platform [8,18]. For the terms with multiple available synonyms, the searching value of each synonym was summed [8,18]. In the search-demand module, the top 10 keyword-related phrases were listed and sorted with their BSI value. The user age, gender, and region distribution data were collected from the demographic portrait module. Therefore, the user demand and user geodemographic data were collected from the Baidu database to analyze users’ demand and public awareness concerning the PE issue.

### Data Analysis 

For each PE topic, the corresponding BSI data were plotted sequentially to describe the trend of public attention. The monthly search index of each PE topic was sorted annually, and the overall time trend change for each domain was determined by the annual percent change (APC) model. This model is designed to examine the overtime change of popularity over a specified fixed interval [19]. The APC was calculated by the Joinpoint Regression model, Program Version 4.7.0.0 (Statistical Research and Applications Branch, National Cancer Institute). A *P*<.05 was considered statistically significant.** **

### Statistical Analysis

The database was constructed with Excel 2019 (Microsoft Corporation). We used Prism 8 for macOS, version 8.4.0 (455; GraphPad Software) to conduct statistical analysis and create figures. 

## Results

### Web-Based Data Trends in PE Topics

We summarized the total BSI of PE-related search keywords in the past 10 years. The retrieved 36 search keywords were manually categorized into four topics, complaint, inquiry, prognosis, and treatment, based on search keywords’ content by the expert panel of medical professionals with over 5 years of experience. The total BSI value of these PE search keywords was 187,558,154. The BSI for each PE topic varied greatly. The search percentage in treatment (n=86,840,487, 46.30%) and complaint (n=66,387,459, 35.40%) accounted for the majority of the PE searches, leaving the inquiry (n=22,320,901, 11.90%) and prognosis (n=12,009,307, 6.40%) topics accounting for less than 20% of the total search (Table 1).

**Figure 1:**
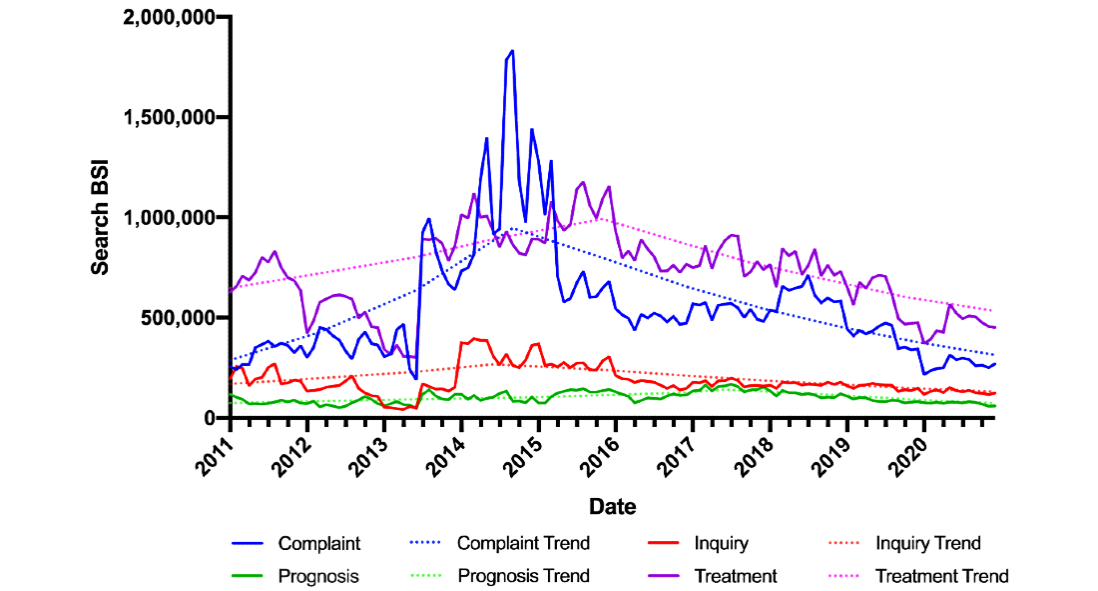
Online interest in premature ejaculation topics over the last 10 years. BSI: Baidu Search Index.

The monthly time series curves of the BSI for each PE topic and the APC trend lines are demonstrated in Figure 1. According to the average count of the annual BSI for each PE topic, these topic trends shared a similar increase for a period of time and redescended at the end of 2020. The following are the overall time average APC for the topics: complaint (1.18%; *P*=.94), inquiry (–2.9%; *P*=.73), prognosis (–0.32%; *P*=.91), and treatment (2.13%; *P*=.65). Specifically, the average APC for the topic complaint was 48.80% (*P*<.001) for 2011 to 2014 and –16.82% (*P*<.001) for 2014 to 2020. The average APC for the topic inquiry was 16.21% for 2011 to 2014 (*P*=.41) and –11.00% (*P*<.001) for 2014 to 2020. For the topic prognosis, the average APC was 11.18% (*P*<.001) from 2011 to 2017 and –19.86% (*P*<.001) from 2017 to 2020. For the topic treatment, the average APC was 14.04% (*P*<.001) from 2011 to 2016 and –38.83% (*P*<.001) from 2016 to 2020. The detailed search trend of the summed searches detailed in each topic are listed in Multimedia Appendix 2.

**Table 1:** Search popularity of each topic in premature ejaculation.

Topic	Baidu Search Index (n=187,558,154), n (%)
Complaint	66,387,459 (35.40)
Inquiry	22,320,901 (11.90)
Prognosis	12,009,307 (6.40)
Treatment	86,840,487 (46.30)

### Geographic Differences

**Figure 2:**
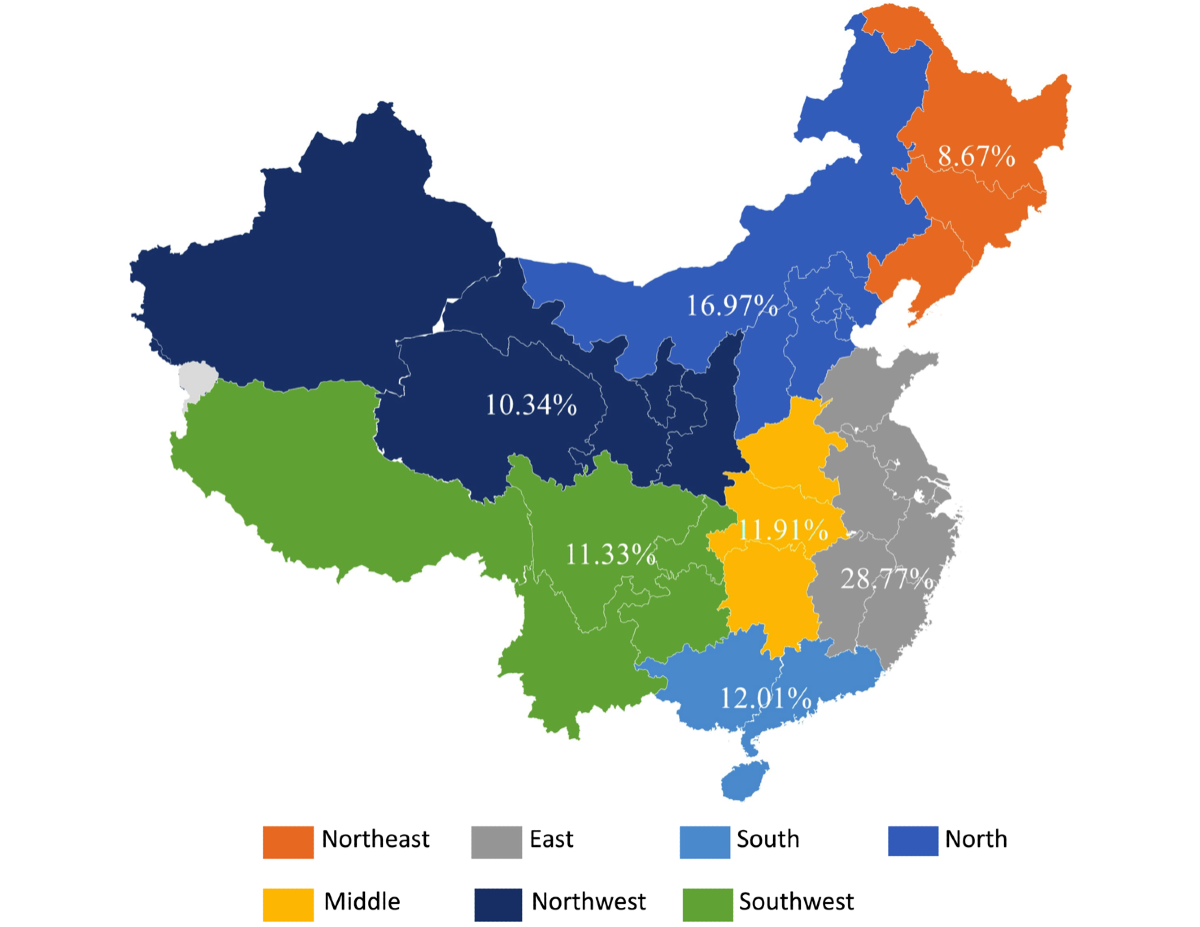
Regional distribution of online interest in premature ejaculation searches over the last 10 years.

The PE BSI geographic distribution was calculated based on provincial data and sorted according to Chinese administrative divisions. The seven regions were as follows: Northeast China, East China, South China, North China, Central China, and Northwest and Southwest China. In Figure 2, the 10-year regional BSI proportions for all PE topics are presented on a map of mainland China with a valid searching record. Notably, people from eastern China (Northeast, East, and South China) made 57.75% (BSI: 108,317,892/187,558,154) of the total search queries. Nevertheless, the queries from west China (Northwest and Southwest China) only made 21.67% (BSI: 40,645,892/187,558,154) of search queries.

### Demographic Differences

From the age distribution (Table 2), in total, it was demonstrated that nearly 70.24% (BSI: 131,732,080/187,558,154) of the PE searches was inquired by the population aged 20 to 39 years (20-29 years and 30-39 years), followed by the population aged 40 to 49 years and younger than 19 years. People older than 50 years accounted for 1.87% (BSI: 3,516,547/187,558,154) of the total popularity. A similar pattern was observed in each PE topic subgroup. With regard to gender differences, the total inquiry was mainly dominated by the male group (Table 3). In detail, the treatment topic was the main concern from the male population, followed by the complaint, prognosis, and inquiry topics.

**Table 2:** Age percentages of search popularity in premature ejaculation topics.

Topic	Baidu Search Index, n (%)				
	≤19 years	20-29 years	30-39 years	40-49 years	≥50 years
General (n=187,558,154)	33,738,405 (17.98)	80,330,533 (42.83)	51,401,547 (27.40)	18,571,122 (9.92)	3,516,547 (1.87)
Complaint (n=66,387,459)	11,590,226 (17.46)	29,134,384 (43.88)	18,625,737 (28.06)	6,048,631 (9.11)	988,481 (1.49)
Inquiry (n=22,320,901)	4,731,811 (21.19)	10,623,644 (47.59)	5,217,263 (23.37)	1,567,679 (7.02)	180,504 (0.81)
Prognosis (n=12,009,307)	3,252,774 (27.08)	4,391,126 (36.56)	3,267,039 (27.20)	975,100 (8.12)	123,268 (1.02)
Treatment (n=86,840,487)	14,163,594 (16.31)	36,181,379 (41.66)	24,291,508 (27.97)	9,979,712 (11.49)	2,224,294 (2.56)

**Table 3:** Gender percentages of search popularity in premature ejaculation topics.

Topic	Female, n (%)	Male, n (%)
General (n=187,558,154)	46,556,413 (24.82)	141,001,741 (75.18)
Complaint (n=66,387,459)	20,435,182 (30.78)	45,952,277 (69.22)
Inquiry (n=22,320,901)	9,144,492 (40.97)	13,176,409 (59.03)
Prognosis (n=12,009,307)	3,928,248 (32.71)	8,081,059 (67.29)
Treatment (n=86,840,487)	13,048,492 (15.03)	73,791,995 (84.97)

### Keyword-Relevant Terms and Search Frequency

**Figure 3:**
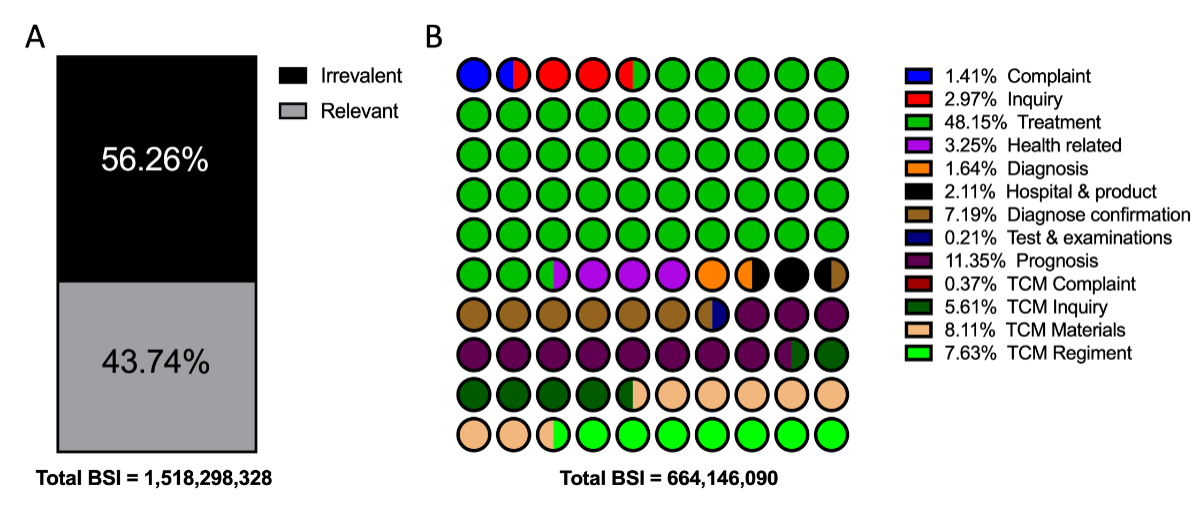
Term categories related to premature ejaculation searches in the Baidu Index demand graph. A: relevant ratio of term BSI; B: distribution of term. BSI: Baidu Search Index; TCM: traditional Chinese medicine.

**Figure 4:**
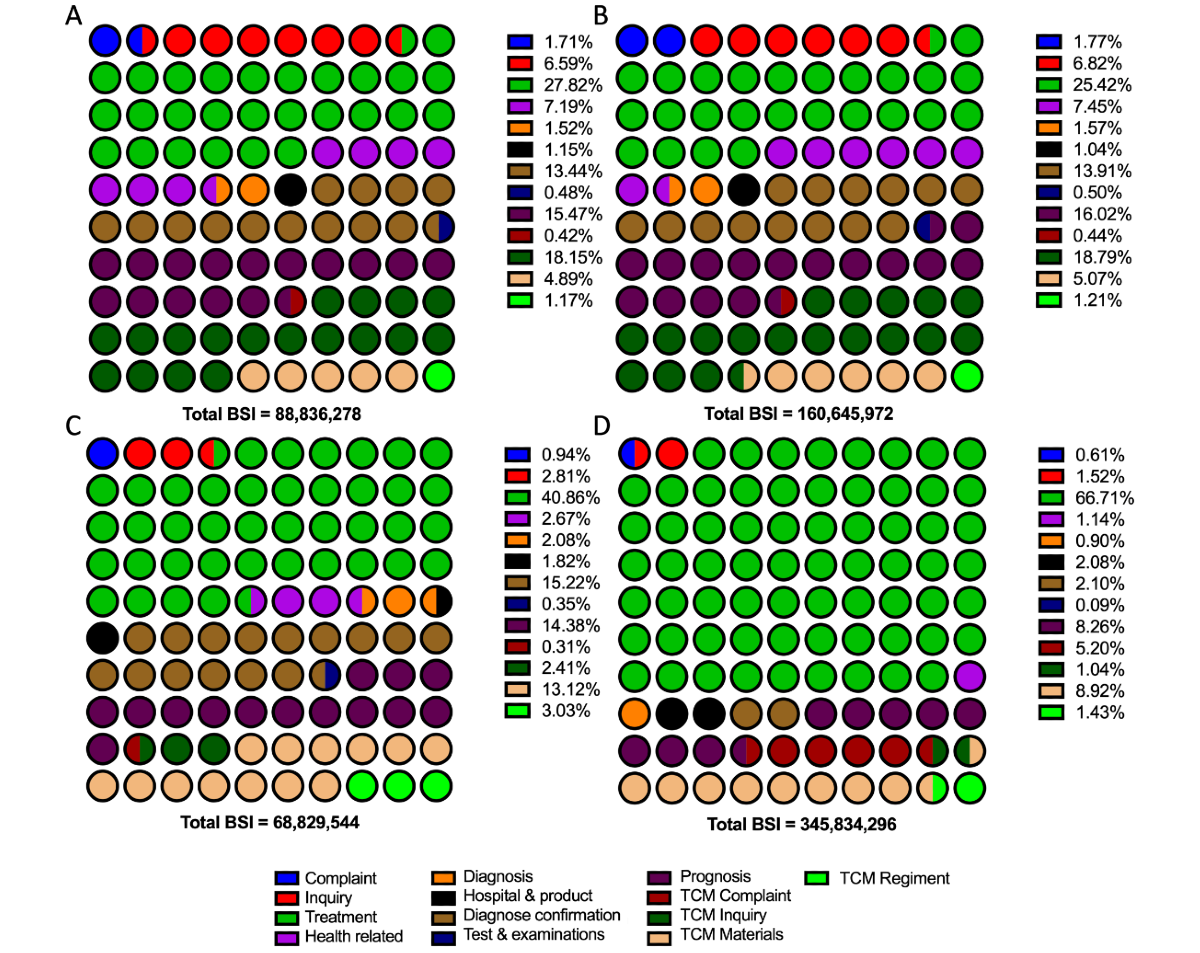
Term categories related to each premature ejaculation topic search in the Baidu Index demand graph. A: topic complaint; B: topic inquiry; C: topic prognosis; D: topic treatment. BSI: Baidu Search Index; TCM: traditional Chinese medicine.

**Figure 5:**
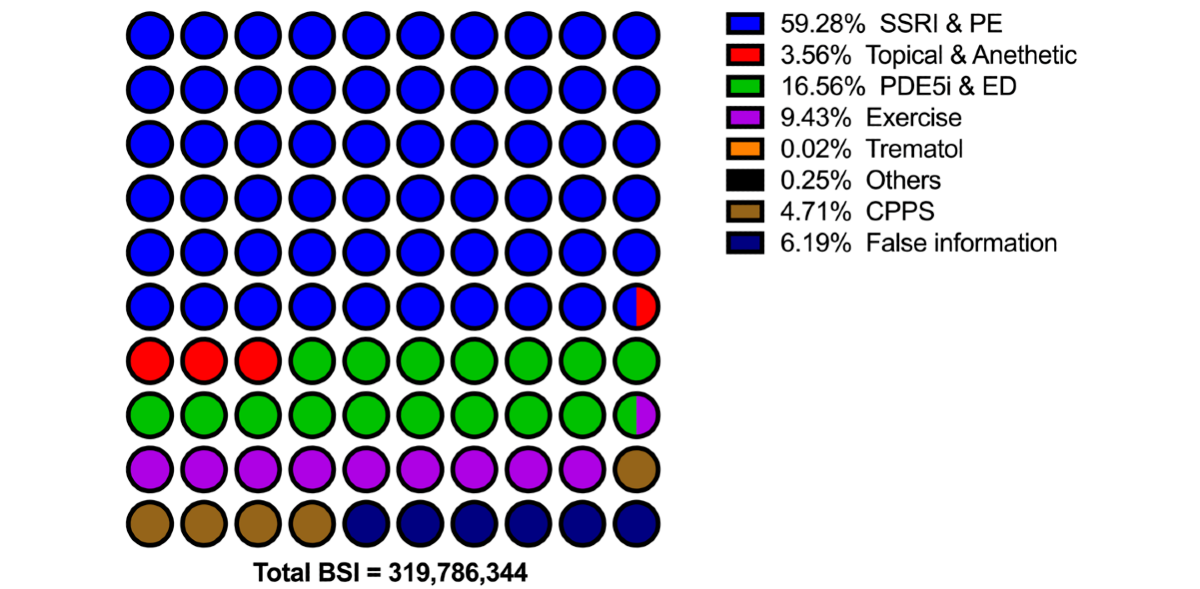
Classification of the detailed options in PE-related treatment search terms. BSI: Baidu Search Index; CPPS: chronic pelvic pain syndrome; ED: erectile dysfunction; PDE5i: phosphodiesterase type 5 inhibitor; PE: premature ejaculation; SSRI: selective serotonin reuptake inhibitor.

In the Baidu Index platform, the user demand data is available for review by sorting the top-searched keyword-relevant terms. The terms were categorized into 14 types to clarify the users’ main concerns and sort their popularity according to the content and implied intention. To better explicitly and comprehensively describe users’ concerns, these categories were defined as (1) irrelevant, (2) complaint, (3) inquiry of etiology, (4) treatment, (5) health-related information, (6) diagnosis, (7) hospital and product, (8) diagnosis confirmation, (9) test and examinations, (10) prognosis, (11) traditional Chinese medicine (TCM) complaint, (12) TCM inquiry, (13) TCM regiment, and (14) TCM materials. The total BSI for the user demand terms was 1,518,298,328, which is nearly 10 times the requests for PE terms. However, only 43.74% (BSI: 664,107,756/1,518,298,328) of the demand search terms were identified as relevant to PE. Detailed distributions of these relative terms were presented in Figure 3. The categorized users’ demand in each PE topic and the detailed treatment inquiries are presented in Figures 4 and 5. Furthermore, the top 3 related terms and their BSI are listed in Multimedia Appendix 3.

## Discussion

### Principal Findings

In investigating PE online interest in mainland China, 36 PE search keywords were identified as valid and could be categorized into the topics complaint, inquiry, prognosis, and treatment. Information on search trend and the geographic, demographic, and user demand information could be identified based on data from the BSI platform.

Baidu is the leading search platform in mainland China. Every day, over billions of user-initiated information queries are made on this platform. With the substantial amount of user-generated data, the medical professionals were enabled with a new insight into users’ information-seeking behaviors and health care problems. Studies have confirmed that the big data from the online platform is capable of assisting with forecasting pandemic outbreaks, identifying population interests, monitoring health education campaigns, and tracking the popularity trend of patients’ preferences [20-22]. Our investigation revealed that the PE search trend on the Baidu Index platform reflects the overall public concern of this health issue.

In our research, 36 PE keywords were categorized into four topic categories. Searches for the complaint topic accounted for over 35% (BSI: 66,387,459/187,558,154) of the search popularity for PE inquiries. This high proportion is probably because the PE search keywords are also words describing symptoms. When it was intuitive and simple to express without content restriction, starting a query with these terms could allow users to decide additional queries depending on the retrieved information. The treatment topic included 21 search keywords, describing PE treatment ranging from medication, surgery, or other available options. From these search keywords, the conveyed definition and concept were restricted and specific. Therefore, this result reveals that Chinese users are likely to assume they have a health problem and are more concerned about the availability and options of solving this problem. Despite the lack of basic understanding of this disease, such as its definition, morbidity, etiology, pathology, or diagnostic criteria, the treatment options were users’ primary concern in health issues like PE. Though six search keywords concerned PE definition and criteria, the total search popularity of the topic inquiry accounted for only 11.90% (BSI: 22,320,901/187,558,154). Hence, a high rate of online self-diagnosis and treatment was observed. Internet self-diagnosis has caused concern about diagnosis delay and misleading treatments [23]. With unproven or biased information that is frequently presented on the internet, the population is given unrealistic expectations. A distrust of professionals with “objective” reasoning could also exacerbate their anxiety about their own problems [24]. Our findings partly represent these issues. Therefore, improving internet health care and optimizing medicine services relies on elevating service quality and popularizing basic medical concepts [25]. 

When evaluating the popularity trend, a similar pattern was observed for the four PE topics. For each topic, the sustained growing trends were only observed in the beginning years. This trend patten reveals that, although the PE incident rate in the population may remain constant, the users’ medical advice-seeking habits may have changed. The medical information from the Baidu platform has always been in public dispute. As the most commonly used search engine in mainland China, Baidu has long been criticized for presenting the top-ranked sites based on higher bids [23]. The biased and inductive information with higher bids are always listed at the top, making high-quality or evidence-based medical information from universities or medical centers more time consuming to obtain [23]. When the Wei Zexi incident broke out in 2016, public critiques of Baidu had reached a peak because the top listed and false advertised treatments claiming to cure synovial sarcoma from this search engine were deemed the leading cause for a 21-year-old student’s death [26]. In 2017, Hu et al [23] found that most health information online from the first 100 links for keywords were generally inadequate. Furthermore, from a recent investigation by Chen et al [27], the content available on Baidu were less credible and inferior to those from Google. Consequently, instead of serving as a search engine that presents information on health education and credible content, Baidu more resembles an advertising platform. Therefore, local public health authorities should participate more and make regulations on the internet service industry, encouraging them to provide more qualified and well-produced information, unbiased information, and evidence-based recommendations on their platform. Hence, the trends in our research indicate that, although the PE prevalence may remain stable, the public awareness may change as people change their information-seeking behavior in response to poor quality content, impacting search popularity.

From the geographic data, we discovered that the PE search popularity was dominated by searches from the East China, North China, and South China regions, where the top four Chinese megacities (Beijing, Shanghai, Guangzhou, and Shenzhen) are located. Although national PE epidemiology data are lacking, this geographic distribution was in line with the leading regional economic level and the demographic distribution [28]. Coinciding with the finding from Xu et al [8] and Wang et al [7], the search popularity’s interregional gap may also be a result of the differences in socioeconomic status, population structure, healthy concepts, and health care policies [9]. Medical professionals could gain insight into the public concern toward PE and customize their clinical session accordingly. 

PE was reported as the most common sexual complaint in males in all age groups in previous epidemiological studies [29]. Though it has long been believed that the PE incidence is correlated with age differences, the existing age PE prevalence data were controversial [30-32]. In our investigation, over 70% of PE online search queries came from males aged 20 to 39 years. This result appears to contradict the Global Online Sexuality Survey, from which the peak PE prevalence was observed in men in their forties [32]. Nevertheless, from an internet-based survey, the highest self-reporting PE incident was for those in their thirties and twenties [30]. The discrepancy between these previous research results may be caused by the screening method, diagnostic standard, and inclusion criteria of risk factors such as age, coitus regularity, circumcision, and masturbation history [31,32]. We believe the openness in Son et al’s [30] investigation resembles the online inquiry on a searching platform. Their discoveries were similar to ours, the results presenting the natural concerns of users and the related popularity distribution [30]. In addition, we noticed the PE inquiry from the female population had reached 24.82% (BSI: 46,556,413) in total and 40.97% (BSI: 9,144,492) in the topic inquiry. Previously, the investigated partners’ satisfaction rates were 45% from the individual with PE’s perspective [33]. According to Zhang et al [34], although only 21.39% of females tended to report PE as hampering sex satisfaction, females are more adversely experienced with relationship issues, such as arousal difficulty, weak sexual desire, and organism inability [34]. Though the cultural differences may underlie searching preference, as the tip of an iceberg, our results may show the concerns and demands of PE issues troubling females. Hence, PE impact on females should be stressed.

In the user demand section, the top 10 most related terms were used for each keyword search. From this section, practitioners were able to gain insight into the patients’ most concerning problems and to confirm individuals’ main intentions from these weekly updated data. Generally, these related terms reflected the wide-ranging scope of concerns when searching PE-related content, such as PE comorbidity, PE training practices, PE etiology, and reproductive influences.

We noticed that the most popular term was “The fastest and easiest way to cure PE?” The high prevalence of this term echoes the topic popularity finding in our research, indicating that the users’ most concerning issue is related to problem solving. Hence, users’ self-diagnosis and self-treatment of issues should be stressed.

The relationship between PE and chronic prostatitis (CP) has been long observed [35]. Suggested as an organic PE cause, the existence of CP in PE is yet to be fully understood. The sexual dysfunction prevalence in males with CP has reached 49% in previous investigations, and the severity of the symptom scoring between these two clinic complaints is well correlated [36,37]. Supporting these investigations, we discovered that “prostate” and “prostatitis” were the most inquired terms from users. This popularity revealed that prostatic problems are the primary concern for patients with PE; also, these people may tend to self-diagnose themselves with prostatitis instead of ejaculatory dysfunction. Whether this concern results from the doctor’s instruction or is based on the patient with PE’s complaint, it is necessary to screen for PE and CP complications.

Additionally, in investigating the users’ demands in treatment, the most queried terms were related to selective serotonin reuptake inhibitors. The followed treatment regimens were phosphodiesterase type 5 inhibitor, topical anesthetic agents, behavior therapy, and tramadol, accounting for over 80% of the search popularity, and these treatment options were either recommended by the sexual dysfunction treatment guidelines or evaluated for safety and efficacy [38,39]. This information could alert the patients to treatment avoidance and contraindication. In addition, better compliance could facilitate patients’ comprehension of the doctor’s instructions.

Nevertheless, terms with false information and their popularity are not noteworthy. These terms typically suggest applying homemade regiments from everyday domestic items on to the penis, ranging from vegetable mixtures with ginger, leeks, or onions to toothpaste or the *Fengyou essence* (a type of essential balm). Though patients could not be harmed much from the vegetable mix remedies aside from allergies or mucosal lesions, damages from the toothpaste and the *Fengyou essence* can be more consequential [6], for the main ingredients of *Fengyou essence* and toothpaste are more abrasive and irritative pharmaceutical materials; the improper application of these regimens with the wrong dose could cause severe skin abrasion or even damage the genital organ [6,40].

Some limitations must be addressed in this study. First, the Baidu Index is only available for search data on the Baidu platform. Though Baidu is the most widely used platform and monopolizes the searching requests in mainland China, the users’ shift to social media for searching is rising. Therefore, lacking a uniform recording system, data from these growing social media sites are not able to be incorporated or be assessed without bias . In addition, in considering the user privacy security, only the user age, gender, and living region information was available for the demographic research. For an in-depth demographic investigation, data such as socioeconomic status, ethnicity, or educational background are required. Additionally, instead of representing the actual search frequency, the BSI is just a weighted index derivative and cannot manifest real-world disease prevalence. Nevertheless, before a nationally conducted PE prevalence and demographic investigation is available, infodemiology research is practical to investigate the PE problems and users’ information-seeking behaviors from the population perspective. 

To our best knowledge, this is the first infodemiology study that investigates PE public concerns in mainland China. We chose to examine the PE popularity because its clinical manifestations are intuitive and easy to describe. More importantly, patients with sexual dysfunction are willing to search online due to privacy concerns. Hence, the real-time updated searching data helps to improve practice standards and policy making for medical professionals and health officials.

### Conclusion

The fluctuating online popularity of PE searches reflects the real-time population demands. It may help medical professionals better understand population interest, population concerns, regional variations, and gender differences on a nationwide scale and make disease-specific health care policies. The internet search data could be more reliable when the insufficient and lagging registry data are completed.

## Appendix

Multimedia Appendix 1 List of searching keywords used in compositing search index.

Multimedia Appendix 2 Supplementary figures of summed search trend and detailed search trend of the premature ejaculation search keywords.

Multimedia Appendix 3 Top 3 terms of users' demands in premature ejaculation.

## Abbreviations

APC: annual percent change
BSI: Baidu Search Index
CP: chronic prostatitis
PE: premature ejaculation
TCM: traditional Chinese medicine
