# Supplementary material for: Online Public Attention Toward Premature Ejaculation in Mainland China: Infodemiology Study Using the Baidu Index
Source: J Med Internet Res. 2021 Aug 26;23(8):e30271. doi: 10.2196/30271 (PMC8430863; doi:10.2196/30271)
Supplement: Multimedia Appendix 1 [file jmir_v23i8e30271_app1.pdf]

Multimedia appendix 1: List of searching keywords used in compositing search index

Domain of terms	Available term in Search engine	English equivilent terms
Complaint	早泄	Premature ejaculation (PE)
	男性早泄	Male PE
	男人早泄	Men PE
	阳痿早泄	PE and impotence
Enquiry	早泄的症状	Symptoms of PE
	什么是早泄	What is PE
	早泄的定义	Definition of PE
	早泄的原因	Cause of PE
	早泄的表现	PE manifestation
	早泄症状	PE symptoms
	早泄原因	PE cause
Prognosis	早泄能治吗	PE treatable
	早泄能治好吗	Is PE curable
	早泄能根治吗	Is PE eradicable
	早泄的危害	Consequence of PE
Treatment	治疗早泄的药物	Medications for PE
	早泄的治疗方法	Treatment for PE
	治早泄的药	Drugs for PE
	治疗早泄的药	PE drug
	治疗早泄	Treat PE
	早泄的药	Medication for PE
	早泄该怎么办	What to do with PE
	早泄药	PE medication
	治早泄	PE treatment
	早泄用药	PE medication
	早泄怎么治疗	How to treat PE
	早泄手术	PE surgery
	如何治疗早泄	How to cure PE
	早泄治疗	Cure PE
	早泄的治疗	PE cure
	早泄怎么治	How to treat with PE condition
	早泄怎么办	How to deal with PE
	早泄如何治疗	How to cure PE problem
	早泄外用药	Externally applied PE regiment
	早泄吃什么药	What medication to take for PE
	怎么治疗早泄	How to treat PE disease

PE, Premature Ejaculation
