# Supplementary material for: Online Public Attention Toward Premature Ejaculation in Mainland China: Infodemiology Study Using the Baidu Index
Source: J Med Internet Res. 2021 Aug 26;23(8):e30271. doi: 10.2196/30271 (PMC8430863; doi:10.2196/30271)
Supplement: Multimedia Appendix 2 [file jmir_v23i8e30271_app2.pdf]

Multimedia Appendix 2. Supplementary figures of summed search trend and detailed search trend of the premature ejaculation search keywords.

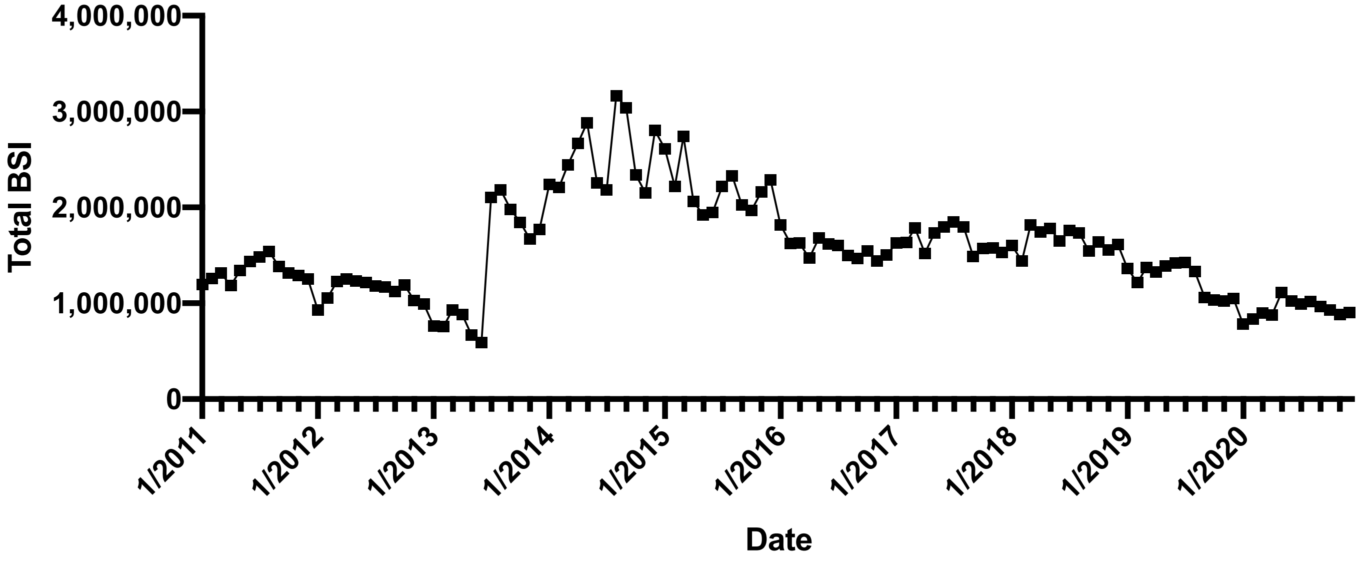

Figure S1-1: Summed Search trend with 36 PE search keywords.

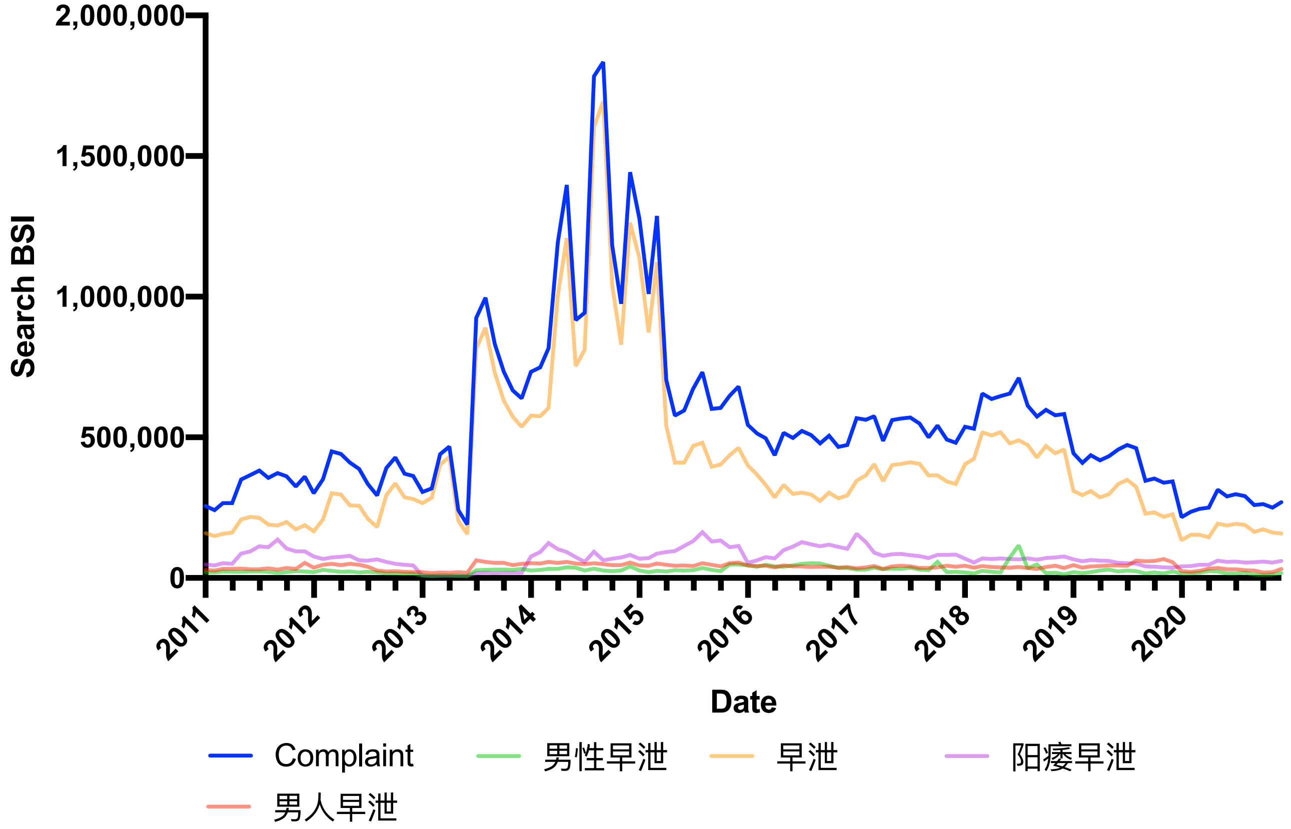

Figure S1-2: Detaled Search trend in PE Complaint topic.

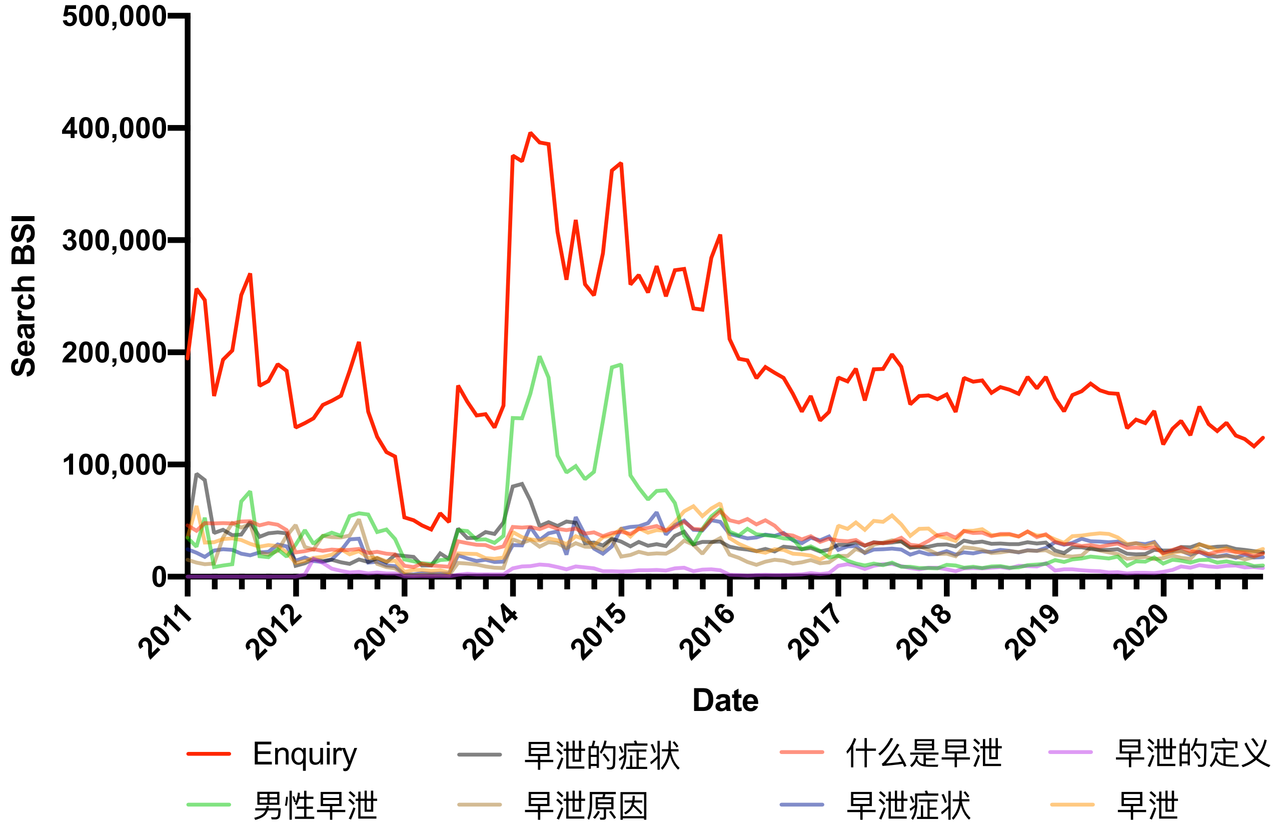

Figure S1-3: Detaled Search trend in PE Enquiry topic.

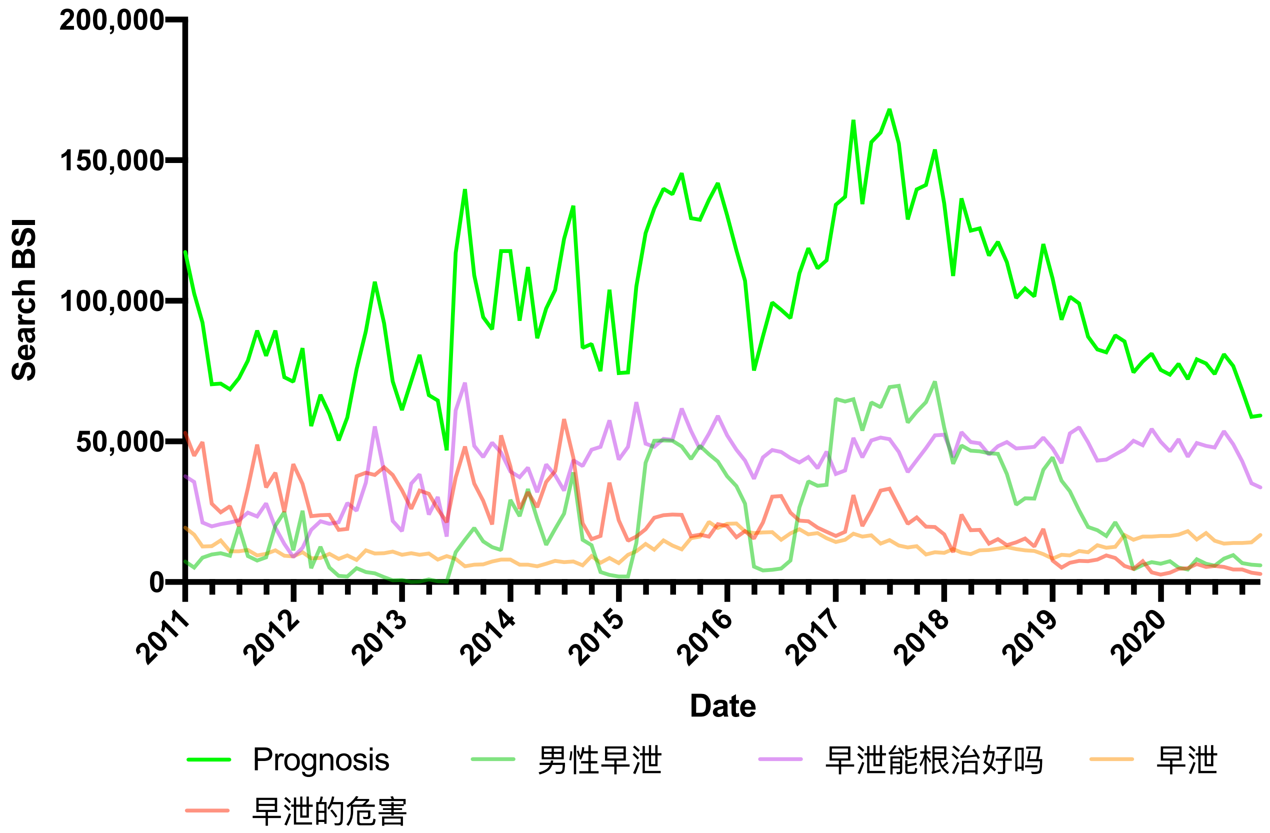

Figure S1-4: Detaled Search trend in PE Prognosis topic.

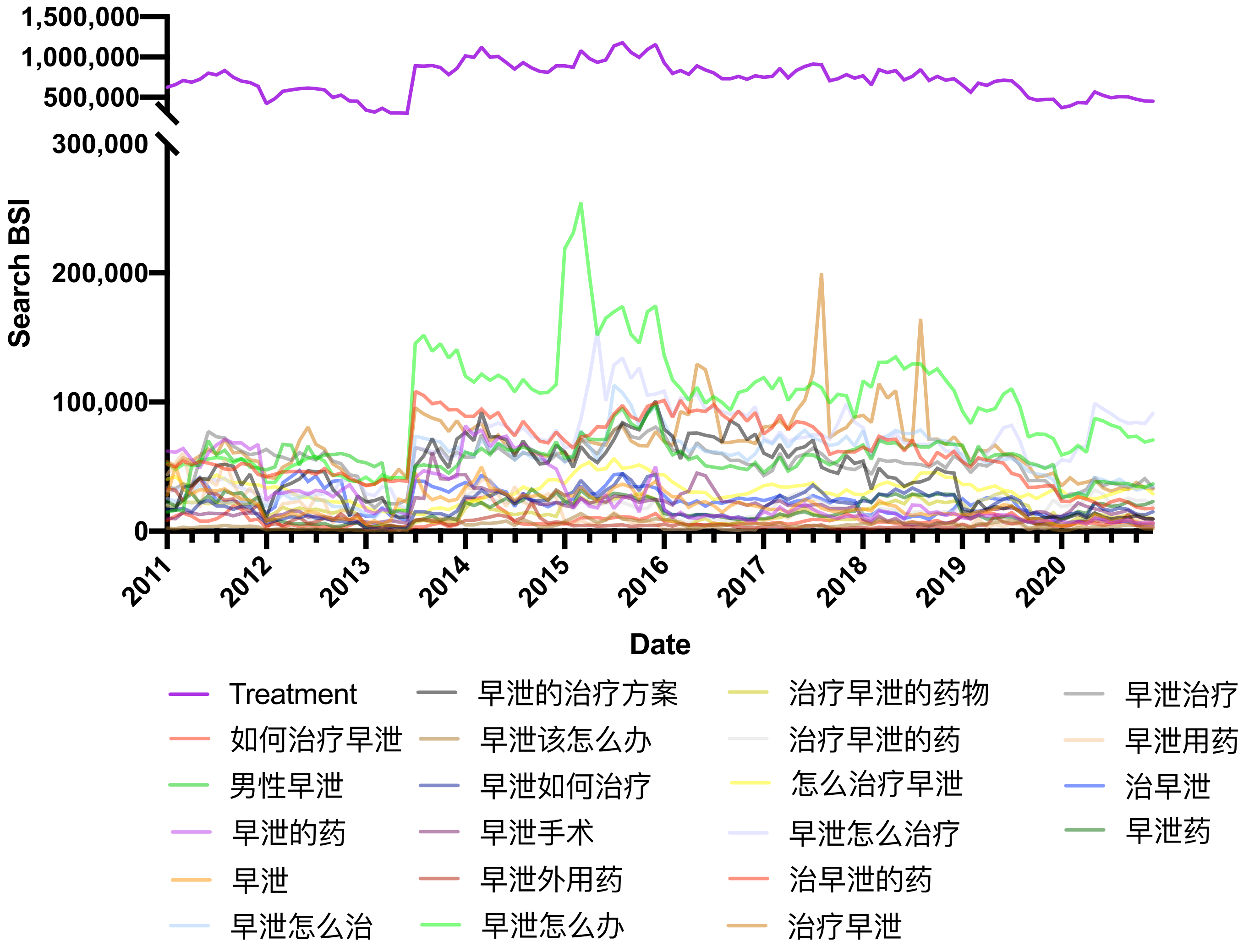

Figure S1-5: Detaled Search trend in PE Treatment topic.
