# Supplementary material for: Online Public Attention Toward Premature Ejaculation in Mainland China: Infodemiology Study Using the Baidu Index
Source: J Med Internet Res. 2021 Aug 26;23(8):e30271. doi: 10.2196/30271 (PMC8430863; doi:10.2196/30271)
Supplement: Multimedia Appendix 3 [file jmir_v23i8e30271_app3.pdf]

Multimedia Appendix 3. Top 3 terms of users' demands in premature ejaculation.

	Term1	BSI	Term2	BSI	Term3	BSI
（2）	遗精	2,005,504	早射	1,298,222	阳萎	676,282
	Spermatorrhea		Early ejaculation (PE)		Impotence/ Erectile dysfunction	
（3）	早射是什么原因引起得	7,224,276	精子变黄了是什么原因	5,491,084	遗精是什么原因引起的	748,962
	What are the cause for early ejaculation (PE)?		What are the cause for yellow semen?		What are the cause for spermatorrhea?	
（4）	治早遲简单快速的方法	53,876,332	必利劲	44,522,070	怎么锻炼治早遲	24,896,278
	Fast and easy way to cure PE		Priligy®		How to cure PE by training?	
（5）	纵欲过度	7,252,986	前列腺	930,304	PC肌	631,626
	Had too much sex		Prostate		Pubococcygeus	
（6）	前列腺炎	7,019,032	尿道炎	382,354	精索静脉曲张	266,176
	Prostatitis		Urethritis		Varicocele	
（7）	阿里巴巴	1,870,848	男科医院	1,458,434	肾宝片	442,118
	Alibaba.com		Andrology hospital		‘SHENBAO’ pill	
（8）	前列腺炎有什么症状	36,037,042	什么是阳萎症状是什么	3,806,104	ED是什么意思	677,708
	What are the symptoms of prostatitis?		What are the symptoms of impotence?		What does ED mean?	
（9）	白细胞高是什么原因	217,520	前列腺按摩	131,864	精子质量标准颜色	128,892
	What causes the white blood cells elevate?		Massage of the prostate.		Normal color of sperm.	
（10）	早些泄能治好吗	28,582,994	手浮八年了会导致不能生育吗	11,742,944	长期过度手浮多久恢复	9,566,606
	Is PE curable?		Is 8-year ‘masterbotion’* causing infertility?		Recovery gap after long term ‘masterbotion’?	
（11）	肾虚	966,952	肾阳虚	564,170	肾阴虚	279,328
	‘Shen’ essence insufficiency		‘Shen’ essence insufficiency of ‘Yang’		‘Shen’ essence insufficiency of ‘Yin’	
（12）	肾虚男的10个表现	24,974,216	肾虚的表现	5,024,508	肾阴虚和肾阳虚的区别	3,040,284
	Top 10 manifestations of men with ‘Shen’ essence insufficiency		Manifestation of ‘Shen’ essence insufficiency		Differences between ‘Shen’ essence insufficiency of ‘Yin’ and ‘Yang’	
（13）	六味地黄丸	14,924,094	金锁固精丸	6,711,060	复方玄驹胶囊	1,523,790
	Six Flavor Rehmanni		Jinsuo Gujing Wan (Golden lock pill to reserve sperm/essence)		Fufang xuanju capsule (Compound Xuanju capsule)	
（14）	早射怎么自我调理	39,755,846	补肾壮阳的最佳方法	3,398,876	男人养肾最佳10食物	1,118,218
	How to recuperate PE by DIY		Best supplementary method to ‘Shen’ and ‘Yang’ essence		Top 10 ‘Shen’ nurturing food for males	

（2）Complaint; （3）Etiology and causes; （4）Treatment and pharmaceutical; （5）Healthcare related terms; （6）Diagnosis; （7）Healthcare services & Commodities; （8）Diagnosis confirmation; （9）Test & Exam; （10）Prognosis; （11）TCM diagnosis; （12）TCM diagnosis confirmation; （13）TCM regiment; （14）TCM remedy and materials;

*Term variety of masturbation for censorship avoidance;

The PE irrelevant terms in category i) were not listed
